# Angio‐associated migratory cell protein promotes colorectal cancer progression by enhancing phosphoglycerate kinase 1 phosphorylation

**DOI:** 10.1002/ccs3.70023

**Published:** 2025-06-16

**Authors:** Wei Zhang, Qian Shi, Qincheng Liu, Haomiao Zhang, Ji Xia, Xueli Zhang

**Affiliations:** ^1^ Fengcheng Hospital of Fengxian District Shanghai China; ^2^ Department of General Surgery Shanghai Fengxian District Central Hospital Shanghai China; ^3^ CAS Key Laboratory of Nutrition, Metabolism and Food Safety Shanghai Institute of Nutrition and Health University of Chinese Academy of Sciences Chinese Academy of Sciences Shanghai China; ^4^ Key Laboratory for Translational Medicine First Affiliated Hospital The First People's Hospital of Huzhou Huzhou University Huzhou China; ^5^ Department of Respiratory and Critical Care Medicine Shanghai East Hospital School of Medicine Tongji University Shanghai China; ^6^ Department of General Surgery Daping Hospital Army Medical University Chongqing China; ^7^ Thoracic Tumor Institute Shanghai Chest Hospital Shanghai China

**Keywords:** Angio‐associated migratory cell protein, colorectal cancer, phosphorylated phosphoglycerate kinase 1, tumor biology, tumor proliferation

## Abstract

To elucidate the oncogenic role of angio‐associated migratory cell protein (AAMP) in colorectal cancer (CRC) and its mechanistic interplay with phosphoglycerate kinase 1 (PGK1). AAMP expression was analyzed in CRC and normal tissues (tissue microarrays‐immunohistochemical/Western blot). Functional impacts were assessed via siRNA knockdown and lentiviral overexpression in CRC cell lines (proliferation: CCK‐8/3‐(4,5‐dimethylthiazol‐2‐yl)‐2,5‐diphenyltetrazolium bromide/clonogenic assays; tumorigenesis: xenografts). Molecular mechanisms were explored through co‐immunoprecipitation, phosphorylation assays, and Ribonucleic Acid (RNA) sequencing. AAMP was significantly upregulated in CRC versus normal tissues (*p* < 0.05), correlating with poor patient survival. AAMP knockdown suppressed CRC cell proliferation, colony formation, and xenograft tumor growth, whereas overexpression exacerbated these phenotypes. Mechanistically, AAMP directly bound PGK1 and enhanced its phosphorylation (p‐PGK1), driving CRC proliferation. PGK1 silencing abrogated AAMP‐mediated proliferative effects. RNA sequencing revealed AAMP modulation of immune‐related pathways (Tumor Necrosis Factor, IL‐17, Jak‐STAT) and key proteins (EGFR, RPL10, NOD2), suggesting dual roles in proliferation. AAMP promotes CRC progression through PGK1 phosphorylation‐dependent metabolic activation, proposing the AAMP‐PGK1 axis as a therapeutic target for advanced CRC.

## Graphical abstract

AAMP promotes CRC progression by enhancing the p‐PGK1, highlighting its novel role in CRC.

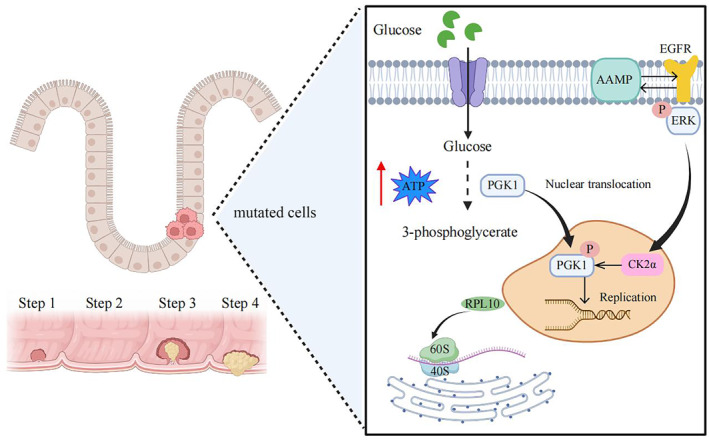

## 1 INTRODUCTION

Colorectal cancer (CRC) is one of the most prevalent and deadly malignancies worldwide, particularly in developed countries, where it ranks as the third leading cause of cancer‐related deaths, following lung and breast cancer.^1^, ^2^, ^3^ Despite advancements in early screening and diagnostic techniques, which have significantly improved early‐stage CRC detection and cure rates, the prognosis for advanced CRC remains poor.^4^ Notably, in patients with distant metastases, despite the use of surgical resection, chemotherapy, and targeted therapy, many patients cannot achieve effective treatment outcomes,^5^ and recurrence and drug resistance remain major issues.^4^ Tumor metastasis, invasion, and drug resistance are the leading causes of CRC‐related mortality,^6^ making treatment particularly challenging. Therefore, a deeper understanding of the molecular mechanisms underlying CRC and identifying new therapeutic targets are crucial to improving patient survival rates and quality of life.

Angio‐associated migratory cell protein (AAMP) is highly expressed in endothelial cells, and recent studies have highlighted its significant role in the initiation and progression of various cancers.^7^, ^8^ AAMP regulates angiogenesis, cell migration, and proliferation and is a pro‐cancer factor in some tumor types.^9^ Studies have shown that AAMP is overexpressed in non‐small cell lung cancer (NSCLC), breast cancer, gastric cancer, and other malignancies^10^ with its high expression closely associated with tumor malignancy, metastatic potential, and patient prognosis.^7^ However, the role of AAMP in CRC remains unclear. Given CRC's high rates of metastasis and recurrence, investigating the function of AAMP in CRC holds significant academic and clinical value.

Recent studies have shown that AAMP not only directly affects tumor growth by influencing processes such as cell proliferation and migration but may also promote tumor development by altering the metabolic state of cancer cells.^7^, ^8^, ^11^ Tumor cells often undergo metabolic reprogramming to meet the demands of rapid growth, with glycolysis being one of the most crucial metabolic pathways.^12^ Phosphoglycerate kinase 1 (PGK1) is a key enzyme in the glycolytic pathway, regulating cellular energy metabolism and proliferation.^13^ Overexpression or aberrant activation of PGK1 is frequently associated with malignant tumor behavior.^13^, ^14^ Previous studies suggest that AAMP may regulate phosphorylated PGK1 through direct interaction, further influencing tumor cell proliferation and metabolic characteristics. Therefore, the interaction between AAMP and PGK1 may play a critical role in the initiation and progression of CRC, although this mechanism has yet to be fully elucidated.

This study aims to explore the biological role of AAMP in CRC and its underlying molecular mechanisms, focusing on the effects of AAMP on CRC cell proliferation, migration, and tumorigenesis, with particular emphasis on the interaction between AAMP and PGK1 and their roles in tumor proliferation and metabolism. The results suggest that AAMP may promote CRC cell proliferation and metabolism by regulating p‐PGK1, facilitating CRC initiation and progression. As a potential target for CRC therapy, AAMP modulation may offer a new strategy, particularly in treating metastatic and drug‐resistant tumors. Investigating AAMP holds promise for improving patient prognosis and may also provide novel biomarkers and targeted drugs for early diagnosis and personalized treatment, advancing the development of precision medicine for CRC.

## 2 MATERIALS AND METHODS

### 2.1 Collection of CRC samples

Ten paired tumor and adjacent normal tissue samples were collected intraoperatively from CRC patients at the First People's Hospital of Huzhou University. Additionally, 195 Formalin‐Fixed, Paraffin‐Embedded samples were obtained from the hospital's pathology department and used to construct tissue microarrays (TMAs). All patients provided written informed consent, and the study was approved by the hospital's ethics committee (Approval No: 2022KYLL018). Fresh tissues were immediately frozen in liquid nitrogen and stored at −80°C for further analysis.

### 2.2 Cell culture

Human CRC cell lines DLD‐1, HCT15, and HT55, as well as the human embryonic kidney cell line 293T, were purchased from the Shanghai Cell Bank of the Chinese Academy of Sciences. DLD‐1, HCT15, and HT55 cells were cultured in RPMI 1640 medium (Gibco) supplemented with 10% fetal bovine serum (FBS, Gibco) and 1% penicillin‐streptomycin (HyClone). 293T cells were cultured in DMEM medium (Gibco) with 10% FBS. All cells were maintained in a 37°C, 5% CO_2_ incubator with medium changes to ensure optimal cell growth. Before experiments, all cells were tested for mycoplasma contamination. Experiments were performed in triplicate to ensure data reliability.

### 2.3 Lentiviral infection and cell transfection

AAMP knockdown cells were constructed using the Plko.1‐shRNA lentiviral system. Two specific shRNAs targeting AAMP were designed: shRNA#1 (GAGATTATCGAGGTGGTAGA) and shRNA#2 (GAGATGGAAGATGTGGACTTT). Additionally, AAMP overexpression cells were generated using the p23‐ZsGreen‐AAMP lentiviral particles. After lentiviral infection, cells were selected with a medium containing 2 μg/mL puromycin (Sangon Biotech) for 3 days to establish stable knockdown or overexpression cell lines. The selected cells were then expanded and used for subsequent experiments.

### 2.4 CCK‐8 assay

Cell proliferation was assessed using the CCK‐8 kit (Dojindo). DLD‐1 and HT55 cells were seeded at a density of 1 × 10^3^ cells/well in a 96‐well plate with six replicates per group. After 24 h of incubation, the experiment was initiated. Every 24 h, 10 μL of CCK‐8 solution was added to each well, and cells were incubated at 37°C for 2 h. The absorbance was measured at 450 nm using a microplate reader (Thermo Fisher). The experiment was performed in triplicate, and data were expressed as the mean ± standard deviation (mean ± SD).

### 2.5 MTT assay

DLD‐1, HT55, and HCT15 cells were seeded at a density of 1 × 10^3^ cells per well in a 96‐well plate with three replicates per group. After culturing for 0, 24, 48, and 72 h, 20 μL of 5 mg/mL 3‐(4,5‐dimethylthiazol‐2‐yl)‐2,5‐diphenyltetrazolium bromide (MTT) solution (Sangon Biotech) was added to each well, and cells were incubated at 37°C for 4 h. The supernatant was discarded, and 200 μL of DMSO (Sangon Biotech) was added to dissolve the formed purple crystals. Absorbance was measured at 490 nm using a microplate reader. The experiment was performed in triplicate, and results are presented as the (mean ± SD).

### 2.6 Crystal violet assay

Cells were seeded at 1 × 10^3^ cells per well in a 6‐well plate, and the experiment was initiated once cells adhered to the surface. The medium was replaced every 24 h, and the cells were cultured for 5–10 days until visible colonies formed. After discarding the medium, cells were washed with Phosphate‐Buffered Saline (PBS) and fixed with 4% paraformaldehyde (Beyotime) for 15 min. The cells were then stained with 0.1% crystal violet solution (Beyotime) for 5 min, and excess dye was removed by washing with PBS. Photographs were taken for record‐keeping, and the dye was dissolved with a 1% Sodium Dodecyl Sulfate (SDS) solution. The absorbance at 570 nm was measured using a microplate reader.

### 2.7 Xenograft tumor study

For the xenograft tumor study, 5‐week‐old male nude mice (purchased from Beijing Vital River Laboratory Animal Technology Co., Ltd.) were used. The study was approved by the Institutional Animal Ethics Committee (Approval No: SINH‐2024‐LLY‐2). DLD‐1 or HT55 cells in the logarithmic growth phase were prepared into a suspension of 5 × 10^6^ cells in 100 μL PBS and injected subcutaneously into both sides of the dorsal area of each mouse. Tumor length and width were measured every 3 days using digital calipers. Tumor volume was calculated using the formula: volume = 0.5 × length × width,^2^ and a tumor growth curve was plotted. After 14 days, the mice were euthanized using CO_2_ asphyxiation. Tumor tissues were excised, weighed, and divided into two portions: one was frozen in liquid nitrogen for molecular analysis, whereas the other was fixed in 4% paraformaldehyde for histological examination.

### 2.8 Western blot analysis

Cells were lysed using Radioimmunoprecipitation Assay Buffer (Beyotime), and total protein was extracted. Protein concentration was quantified using the Bicinchoninic Acid method (Beyotime). A 50 μg protein was separated by SDS‐Polyacrylamide Gel Electrophoresis (PAGE) and transferred to a Polyvinylidene Fluoride membrane (Millipore). The membrane was incubated with AAMP (Proteintech), epidermal growth factor receptor (EGFR) (Proteintech), RPL10 (Proteintech); NOD2 (Proteintech), and Glyceraldehyde 3‐Phosphate Dehydrogenase (Proteintech) primary antibodies to detect the target proteins. Detection was performed using the Enhanced Chemiluminescence chemiluminescence reagent (Thermo Fisher), and images were analyzed using the Image Lab software.

### 2.9 Co‐immunoprecipitation

Flag‐tagged AAMP overexpression 293T cells were lysed, and the lysates were incubated with Flag antibody (Sigma‐Aldrich) and protein A/G magnetic beads (Beyotime). The immune complexes were separated by SDS‐PAGE and analyzed by Western blot to confirm the interaction with PGK1.

### 2.10 RNA sequencing and bioinformatics analysis

Total Ribonucleic Acid (RNA) was extracted from sh‐AAMP and control DLD‐1 cells using the TRIzol reagent (Invitrogen). RNA integrity was assessed, and sequencing libraries were constructed. High‐throughput sequencing was performed on the Illumina NovaSeq 6000 platform. Differentially expressed genes (DEGs) were identified using DESeq2 (|log_2_FC| > 1, *p*adj < 0.05). Gene Ontology (GO) and Kyoto Encyclopedia of Genes and Genomes (KEGG) analyses were conducted using the Metascape platform. The Search Tool for the Retrieval of Interacting Genes/Proteins (STRING) database (http://string‐db.org) was also used to construct the AAMP‐associated protein interaction network.

### 2.11 Mass spectrometry analysis

Flag‐tagged 293T cells were lysed, and the target protein was enriched using co‐immunoprecipitation (Co‐IP). Differential bands were visualized with a silver staining kit (Beyotime) and sent to Applied Protein Technology for Liquid Chromatography–Mass Spectrometry/MS mass spectrometry analysis and identification.

### 2.12 Statistical analysis

All experimental data were analyzed using GraphPad Prism 10 software. Data are presented as the mean ± SD. Comparisons between two groups were performed using *t*‐tests, whereas comparisons among multiple groups were analyzed using one‐way Analysis of Variance. A significance level of *p <* 0.05 was considered statistically significant. Each experiment was repeated at least three times to ensure data reliability.

## 3 RESULTS

### 3.1 The expression of AAMP is significantly upregulated in CRC, suggesting its potential role in tumor progression

**FIGURE 1:**
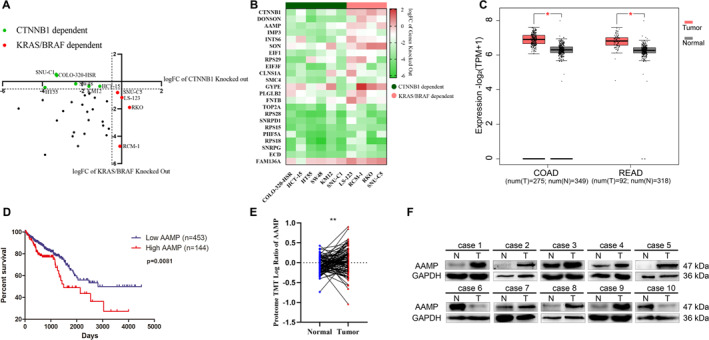
Upregulation of angio‐associated migratory cell protein (AAMP) in colorectal cancer (CRC) and its association with poor prognosis in patients. (A) CTNNB1 and KRAS/BRAF cell line classification schematic diagram. (B) Heatmap of CTNNB1 and KRAS/BRAF cell line dependency. (C) Analysis of AAMP expression levels in various tumors from the TCGA database. (D) Kaplan–Meier survival curve analysis comparing cumulative survival rates of 597 CRC patients from the TCGA cohort with high AAMP expression (*n* = 144) and low AAMP expression (*n* = 453), using the log‐rank test for significance (*p* = 0.0001). (E) Analysis of AAMP protein expression in tumor and normal tissues from the clinical proteomic tumor analysis consortium database with normal (*n* = 62) and tumor (*n* = 318) tissue sample sizes. (F) Western blot analysis of AAMP protein expression in paired tumor (T) and adjacent normal (N) tissues from CRC patients. Sample size: *n* = 10 pairs.

Clustered Regularly Interspaced Short Palindromic Repeats (CRISPR) screening data from the project score database identified CTNNB1 as a key gene in APC‐dependent CRC cell lines, whereas Kirsten Rat Sarcoma Viral Oncogene Homolog (KRAS) and B‐Raf Proto‐Oncogene, Serine/Threonine Kinase (BRAF) were predominant in KRAS/BRAF‐dependent lines (Figure 1A). Heatmap analysis revealed several genes significantly enriched in APC‐dependent cell lines including AAMP, CTNNB1, and Downstream Neighbor of SON (DONSON) (Figure 1B). The Cancer Genome Atlas (TCGA) data showed that AAMP expression was upregulated across multiple cancer types including cholangiocarcinoma, diffuse large B‐cell lymphoma, glioblastoma multiforme, brain lower grade glioma, liver hepatocellular carcinoma, pancreatic adenocarcinoma, testicular germ cell tumors, and thymoma. Notably, AAMP expression was significantly higher in colon adenocarcinoma and rectal adenocarcinoma tissues compared to normal controls (Figure 1C, Figure S1). Kaplan–Meier survival analysis indicated that patients with high AAMP expression had significantly poorer overall survival than those with low expression (*p* = 0.002, Figure 1D). Clinical proteomic tumor analysis consortium (CPTAC) proteomic data further confirmed elevated AAMP protein levels in CRC tissues relative to normal tissues (Figure 1E). Western blot analysis of 10 paired CRC tumors and adjacent normal tissues collected in this study consistently showed significantly increased AAMP protein expression in tumor samples (Figure 1F).

### 3.2 AAMP may play a role in colorectal cancer by regulating cell proliferation

**FIGURE 2:**
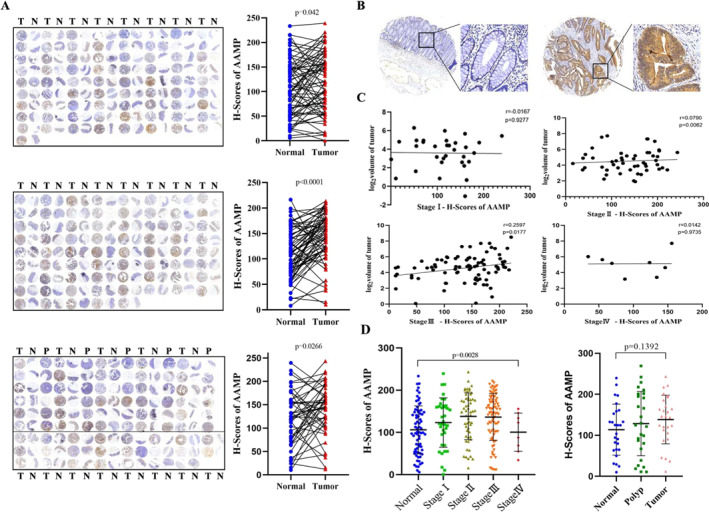
Expression analysis of angio‐associated migratory cell protein (AAMP) in colorectal cancer (CRC) and its correlation with clinical characteristics. (A) Immunohistochemical (IHC) staining analysis of AAMP on a tissue microarray consisting of 195 paired CRC and adjacent normal tissue samples. (B) Scoring of signal intensity for each tissue core using the Vectra 2 system. Scale bar: 50 μm. (C) Linear regression analysis of AAMP H‐scores and tumor volume across different CRC stages. (D) IHC‐based AAMP expression scores, comparing AAMP expression between CRC tissues, adjacent normal tissues, and healthy controls. Analysis of AAMP expression levels across different tumor stages (I–IV) in CRC patient samples (*n* = 195). Statistical significance was assessed using the Kruskal–Wallis test.

To assess AAMP expression in CRC, immunohistochemical (IHC) staining was performed on TMAs comprising 195 paired primary CRC and adjacent normal tissues. Each tissue core was scored using the Vectra 2 system. IHC analysis revealed that AAMP expression was significantly higher in CRC tissues than adjacent normal tissues (Figure 2A,B, *p <* 0.05). Linear regression analysis revealed a significant positive correlation between AAMP H‐scores and tumor volume in Stage II (*p* = 0.0062) and Stage III (*p* = 0.0177) CRC patients but not in Stage I or Stage IV (Figure 2C). Furthermore, analysis of CRC tissues at different tumor stages (Figure 2D) demonstrated a significant correlation between AAMP expression levels and tumor staging (Kruskal–Wallis test, *p* > 0.05), suggesting that AAMP may be involved in CRC proliferation and malignant progression. Subsequent analysis of clinical and pathological parameters indicated that high AAMP expression was significantly correlated with Ki67 status (*p* = 0.0057) and patient age (*p* = 0.006), but not with lymph node metastasis status (*p* = 0.7386) (Table 1).

**TABLE 1:** Correlation between AAMP and clinicopathological features in colorectal cancer tissues.

Clinicopathological factor	Total	AAMP H‐S (0–6)	AAMP H‐S (7–12)	*χ* ^2^	*p*‐value
Sex					
Male	118	57	61	0.6802	0.4095
Female	78	33	45		
Age					
≤65 years	86	49	37	7.546	0.0060**
>65 years	110	41	69		
Tumor size					
<5 cm	114	51	63	0.3044	0.5811
≥5 cm	80	39	41		
Ki67					
<0.8	88	50	38	7.64	0.0057**
≥0.8	108	40	68		
Lymph node metastasis					
No	102	48	54	0.1114	0.7386
Yes	94	42	52		

Abbreviation: AAMP, Angio‐associated migratory cell protein.

** *p* < 0.01.

### 3.3 AAMP promotes CRC cell growth in vitro by regulating cell proliferation

**FIGURE 3:**
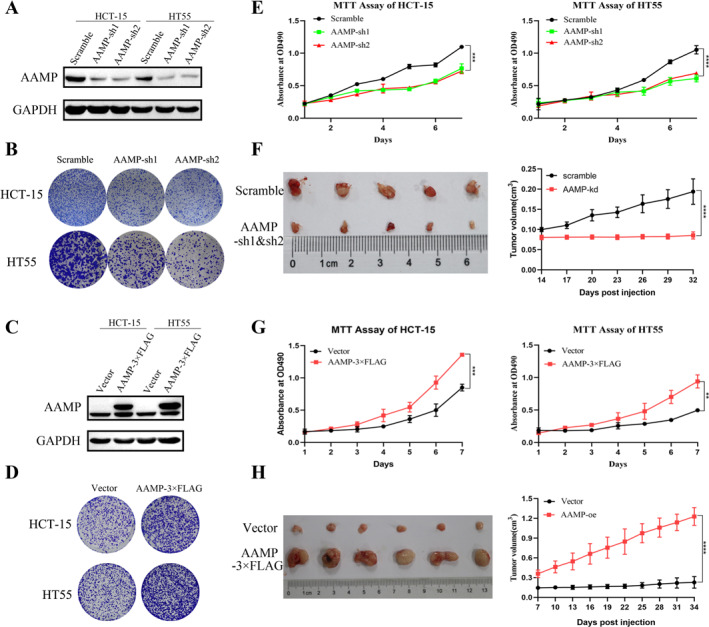
Angio‐associated migratory cell protein (AAMP) regulates colorectal cancer cell proliferation and colony formation in vitro through knockdown or overexpression. (A) Western blot analysis showing AAMP knockdown efficiency in HCT‐15 and HT55 cells. (B) Crystal violet staining assay assessing the impact of AAMP knockdown on colony formation in HCT‐15 and HT55 cells. (C) Western blot analysis showing AAMP overexpression efficiency in HCT‐15 and HT55 cells. (D) Crystal violet staining assay evaluating the effect of AAMP overexpression on colony formation in HCT‐15 and HT55 cells. (E) 3‐(4,5‐dimethylthiazol‐2‐yl)‐2,5‐diphenyltetrazolium bromide (MTT) assay measuring the effect of AAMP knockdown on cell proliferation in HCT‐15 and HT55 cells with absorbance measured at 570 nm. (F) Tumor imaging and size comparison from mice implanted with scramble control or AAMP knockdown HT55 cells (*n* = 6). (G) MTT assay assessing the effect of AAMP overexpression on cell proliferation in HCT‐15 and HT55 cells with absorbance measured at 570 nm. (H) Tumor imaging and size comparison from mice implanted with vector control or AAMP overexpression HT55 cells (*n* = 6). Data are presented as means ± SD with three independent experiments performed. **p < 0.01, ***p < 0.001, ****p < 0.0001.

To investigate the role of AAMP in CRC cell proliferation, AAMP was knocked down in DLD‐1 and HT55 cells using shRNA. MTT and crystal violet assays significantly reduced cell proliferation and colony formation in AAMP‐depleted cells compared to controls (*p* < 0.05; Figure 3A,B,E). Conversely, AAMP was stably overexpressed in HT55 and HCT15 cells. MTT and colony formation assays showed significantly enhanced proliferative capacity upon AAMP overexpression (*p* < 0.05; Figure 3C,D,G). In vivo, AAMP knockdown in HT55 cells significantly suppressed tumor growth in a nude mouse xenograft model (*p* < 0.05; Figure 3F). In contrast, AAMP overexpression significantly accelerated tumor growth in xenografts derived from HT55 cells (*p* < 0.05; Figure 3H).

### 3.4 AAMP enhances CRC cell proliferation and growth by promoting p‐PGK1

**FIGURE 4:**
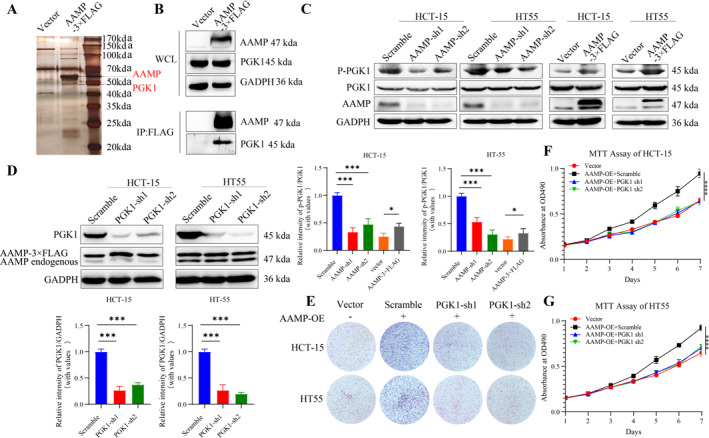
Angio‐associated migratory cell protein (AAMP) promotes colorectal cancer cell proliferation and growth by interacting with phosphoglycerate kinase 1 (PGK1). (A) Silver‐stained gel showing the interaction between AAMP and PGK1, comparing AAMP overexpressing samples with controls. (B) Co‐IP assay detecting the direct interaction between AAMP and PGK1 using HT55 cell lysates. (C) Western blot analysis of PGK1 and p‐PGK1 levels in scramble control and AAMP knockdown HCT15 and HT55 cells as well as in vector control and AAMP overexpressing HT55 and HCT15 cells. (D) Western blot analysis showing PGK1 knockdown efficiency in AAMP overexpressing HT55 cells using Glyceraldehyde 3‐Phosphate Dehydrogenase as an internal control. (E) Crystal violet staining assay evaluating the effect of PGK1 knockdown on colony formation in vector control and AAMP overexpressing HT55 cells with data quantified using ImageJ software. (F) 3‐(4,5‐dimethylthiazol‐2‐yl)‐2,5‐diphenyltetrazolium bromide (MTT) assay assessing the effect of PGK1 knockdown on cell proliferation in vector control and AAMP overexpressing HCT15 and HT55 cells with absorbance measured at 570 nm. (G) MTT assay evaluating the effect of PGK1 knockdown on cell proliferation in vector control and AAMP overexpressing HCT15 cells with absorbance measured at 570 nm. Experimental data are presented as means ± SD. ***p < 0.001, ****p < 0.0001.

Mass spectrometry analysis was performed to investigate the mechanism by which AAMP promotes CRC cell proliferation and identified PGK1 as a potential AAMP‐interacting protein. This interaction was further validated by Co‐IP assays (Figure 4A,B). PGK1, a rate‐limiting enzyme in glycolysis, is known to play a key role in cell proliferation. Western blot analysis showed that AAMP knockdown significantly reduced phosphorylated PGK1 (p‐PGK1) levels in DLD‐1 and HT55 cells, while AAMP overexpression increased p‐PGK1 levels in HT55 and HCT15 cells (Figure 4C). To further confirm the regulatory role of AAMP on PGK1, PGK1 was knocked down in AAMP‐overexpressing HT55 and HCT15 cells. The results demonstrated that PGK1 knockdown significantly suppressed the enhanced proliferation and colony formation induced by AAMP overexpression (Figure 4D–G, *p* < 0.05).

### 3.5 AAMP regulates CRC cell function through immune‐related pathways and key protein networks

**FIGURE 5:**
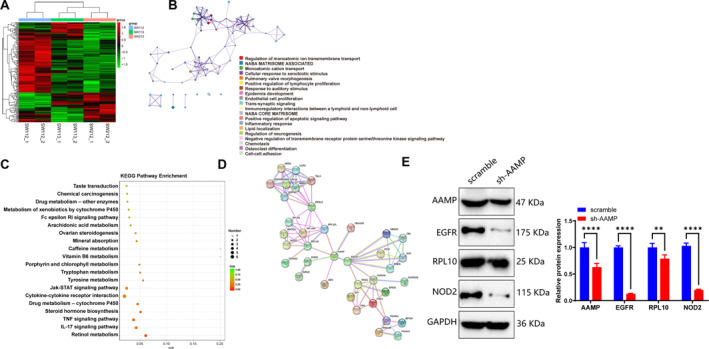
Gene network and bioinformatics analysis of angio‐associated migratory cell protein (AAMP). (A) Heatmap of differentially expressed genes (DEGs) between the scramble control and sh‐AAMP groups. The heatmap color indicates changes in gene expression with light red representing upregulated genes and green representing downregulated genes. The filtering criteria were |log_2_FC| > 1 and *p*adj < 0.05. (B) Gene ontology (GO) enrichment analysis of DEGs performed using the Metascape platform, with GO terms categorized by biological process. (C) Kyoto Encyclopedia of Genes and Genomes (KEGG) pathway enrichment analysis, where the *x*‐axis represents −log_10_ (*p*‐value) and the *y*‐axis represents KEGG pathways. The bubble size reflects the number of genes involved in each pathway, whereas the bubble color indicates the significance level (*p*‐value). (D) Protein–protein interaction network analysis of AAMP‐related proteins using the Search Tool for the Retrieval of Interacting Genes/Proteins database with a confidence threshold set at 0.7. (E) Western blot analysis of epidermal growth factor receptor, RPL10, and NOD2 protein expression levels in HCT15 cells between the scramble control group and the AAMP knockdown group.

To further explore the underlying mechanisms of AAMP in CRC, RNA sequencing was performed on transduced HCT‐15 cells. A total of 141 DEGs were identified between the sh‐AAMP and scramble control groups (|log_2_FC| > 1, Padj <0.05; Figure 5A). GO enrichment analysis revealed that AAMP knockdown significantly affected biological processes related to angiogenesis and innate immune response (Figure 5B). KEGG pathway analysis further indicated that DEGs were significantly enriched in cytokine‐cytokine receptor interaction, Tumor Necrosis Factor signaling pathway, IL‐17 signaling pathway, and Jak‐STAT signaling pathway (Figure 5C).

STRING protein‐protein interaction network analysis identified several AAMP‐associated proteins, including PGK1 (glycolysis), EGFR, RPL10 (ribosomal protein), and NOD2 (innate immunity), suggesting a broader regulatory role for AAMP in CRC proliferation and immune modulation (Figure 5D). Western blot analysis confirmed that AAMP knockdown reduced the expression of EGFR, RPL10, and NOD2 (Figure 5E). These findings demonstrate that, in addition to regulating PGK1, AAMP may influence key immune and proliferative pathways.

## 4 DISCUSSION

This study reveals the pro‐tumorigenic role of AAMP in CRC and systematically evaluates its expression in clinical tissues using TMA technology, further confirming its correlation with poor patient prognosis. The results indicate that AAMP expression is significantly higher in CRC tissues compared to normal tissues, and its high expression is closely associated with shortened patient survival and tumor recurrence, suggesting that AAMP may serve as a potential biomarker for CRC. This finding aligns with observations in other cancer types, particularly in NSCLC and breast cancer, where AAMP has been identified as an important oncogenic factor.^7^, ^10^, ^15^ Although the oncogenic role of AAMP has been recognized in several cancers, its function in CRC has not been systematically studied. This study fills this research gap and provides new theoretical evidence supporting AAMP as a potential therapeutic target.

This study demonstrates that AAMP enhances CRC cell proliferation by directly binding to PGK1 and promoting phosphorylation. PGK1, a key rate‐limiting enzyme in glycolysis, plays a critical role in cancer cell metabolic adaptation and survival through phosphorylation‐dependent mechanisms. AAMP may regulate PGK1 activity indirectly via modulation of AMP‐Activated Protein Kinase signaling, thereby increasing glycolytic capacity in CRC cells. PGK1 has also been reported to regulate the diversion of glycolytic intermediates into the pentose phosphate pathway,^16^ contribute to serine biosynthesis,^17^ and influence mitochondrial function.^18^ Alterations in PGK1 activity can affect the generation of 3‐phosphoglycerate, impacting serine metabolism and mitochondrial homeostasis through changes in cellular energy states. These findings suggest that AAMP promotes metabolic reprogramming in CRC via PGK1 phosphorylation, offering a novel perspective on tumor metabolic regulation and identifying AAMP as a potential target for metabolic‐based therapy.

Compared to previous studies,^7^, ^15^, ^19^ this research expands the understanding of AAMP's role in tumorigenesis and progression. In NSCLC and breast cancer, AAMP primarily promotes cell proliferation via the Akt signaling pathway or enhances cell migration through epithelial‐mesenchymal transition (EMT). However, in CRC, our findings indicate that AAMP does not significantly activate Akt or EMT‐related signaling but instead promotes tumor cell proliferation primarily through PGK1 phosphorylation and glycolysis regulation. This discovery suggests that AAMP exhibits functional specificity across different cancer types, with its oncogenic role in CRC is more dependent on metabolic adaptation than traditional proliferation signaling pathways.^20^

Furthermore, PGK1, a key metabolic regulator, has been widely studied in various cancers.^21^ In hepatocellular carcinoma and glioblastoma, PGK1 phosphorylation has been shown to facilitate glycolytic reprogramming, enhancing tumor cell adaptation to energy demands.^22^, ^23^, ^24^ However, the specific role of PGK1 in CRC metabolic regulation has not been systematically investigated. This study is the first to reveal that AAMP promotes CRC cell proliferation through PGK1‐mediated metabolic adaptation, offering new insights into PGK1 as a metabolic target for CRC and suggesting that AAMP may act as an upstream regulator of PGK1 phosphorylation.

Based on CRISPR screening data, AAMP was identified as a functionally relevant gene in APC‐dependent CRC cell lines. AAMP plays critical roles in various physiological and pathological processes^25^ and has been associated with tumor progression, metastasis, and poor prognosis across multiple cancer types.^15^ In NSCLC, AAMP interacts with EGFR, enhancing its phosphorylation and activating the ERK1/2 pathway, thereby promoting proliferation and resistance to chemotherapy.^10^ Although genes such as CTNNB1 and DONSON have been extensively studied in CRC,^26^, ^27^, ^28^ the role of AAMP in this context remains poorly characterized. Multi‐omics analyses, including TCGA and CPTAC datasets, revealed that AAMP is significantly upregulated in CRC tissues. This finding was further validated by paired tissue analysis and TMA studies. Kaplan–Meier survival analysis indicated a significant association between high AAMP expression and poor prognosis, suggesting its potential as a prognostic biomarker.

Importantly, the data revealed that AAMP promotes metabolic adaptation in CRC cells by regulating PGK1 phosphorylation, in contrast to traditional oncogenic drivers, such as Epidermal Growth Factor and Vascular Endothelial Growth Factor, which primarily act through growth factor receptor signaling.^29^, ^30^ This mechanistic distinction highlights AAMP as a potentially unique metabolic regulatory target. Additionally, previous studies have shown that AAMP regulates endothelial cell migration and angiogenesis via the RhoA/Rho‐Associated, Coiled‐Coil Containing Protein Kinase pathway,^11^ though its role in CRC‐associated angiogenesis requires further investigation. The oncogenic function of AAMP in CRC was confirmed through TMA analysis, Western blotting, and functional assays. Large‐scale evaluation using clinical tissue samples enhances the translational relevance of these findings. Targeting AAMP and its regulatory axis, such as PGK1, with small‐molecule inhibitors may offer a novel strategy for metabolic therapy in CRC.

Despite these insights, this study has certain limitations. First, the specific phosphorylation sites and molecular mechanisms by which AAMP regulates PGK1 remain unclear, requiring further biochemical and structural biology analyses. Second, most experiments were conducted in vitro, and the impact of AAMP‐mediated PGK1 phosphorylation on tumor growth has yet to be validated in vivo using animal models. Future studies should incorporate mouse models to further confirm these findings. Additionally, the sample size of patient tissues is limited which may affect the statistical power and broader applicability of the results. Expanding the patient cohort will be essential to further evaluate AAMP's clinical potential as a CRC prognostic biomarker.

Future research should also explore whether AAMP‐mediated PGK1 phosphorylation influences other metabolic pathways and assess its potential as a target for combination therapies. By elucidating the molecular mechanisms underlying AAMP's role in CRC metabolic regulation and developing targeted inhibitors, this study may contribute to precision treatment strategies for CRC (see Graphical abstract).

## 5 CONCLUSION

This study reveals the biological role and oncogenic potential of AAMP in CRC through detailed experimental methods. The results demonstrate that AAMP expression is significantly elevated in CRC tissues compared to normal tissues, and its high expression is closely associated with poor prognosis. Experimentally, downregulating AAMP expression significantly inhibits CRC cell growth and tumor formation, whereas overexpressing AAMP enhances these tumor characteristics. Furthermore, the interaction between AAMP and PGK1 and the increased p‐PGK1 is a key mechanism by which AAMP promotes CRC cell proliferation. Therefore, the regulation of AAMP highlights its critical oncogenic role in CRC and suggests that it could serve as an effective therapeutic target for CRC treatment.

## AUTHOR CONTRIBUTIONS

W.Z., Q.S., and Q.L. contributed equally to this work and share co‐first authorship. W.Z. and X.Z. conceived and designed the study. Q.S., Q.L., and H.Z. performed the experiments and data acquisition. J.X. and H.Z. conducted data analysis and interpretation. X.Z. supervised the overall project and secured funding. W.Z., Q.S., and Q.L. drafted the manuscript. All authors reviewed and approved the final version of the manuscript.

## CONFLICT OF INTEREST STATEMENT

The authors declare no conflicts of interest.

## ETHICS STATEMENT

This study was approved by the Clinical Ethics Committee of Central Laboratory, First Affiliated Hospital of Huzhou University (No. 2022KYLL018). All animal experiments were approved by the Animal Ethics Committee of CAS Key Laboratory of Nutrition, Metabolism and Food Safety, Shanghai Institute of Nutrition and Health, University of Chinese Academy of Sciences, Chinese Academy of Sciences (No. SINH‐2024‐LLY‐2). Clinical trial number: not applicable.

## Supporting information

Figure S1

## Data Availability

All data can be provided as needed.
