# Supplementary figures and images for: Angio‐associated migratory cell protein promotes colorectal cancer progression by enhancing phosphoglycerate kinase 1 phosphorylation
Source: J Cell Commun Signal. 2025 Jun 16;19(2):e70023. doi: 10.1002/ccs3.70023 (PMC12170457; doi:10.1002/ccs3.70023)

**
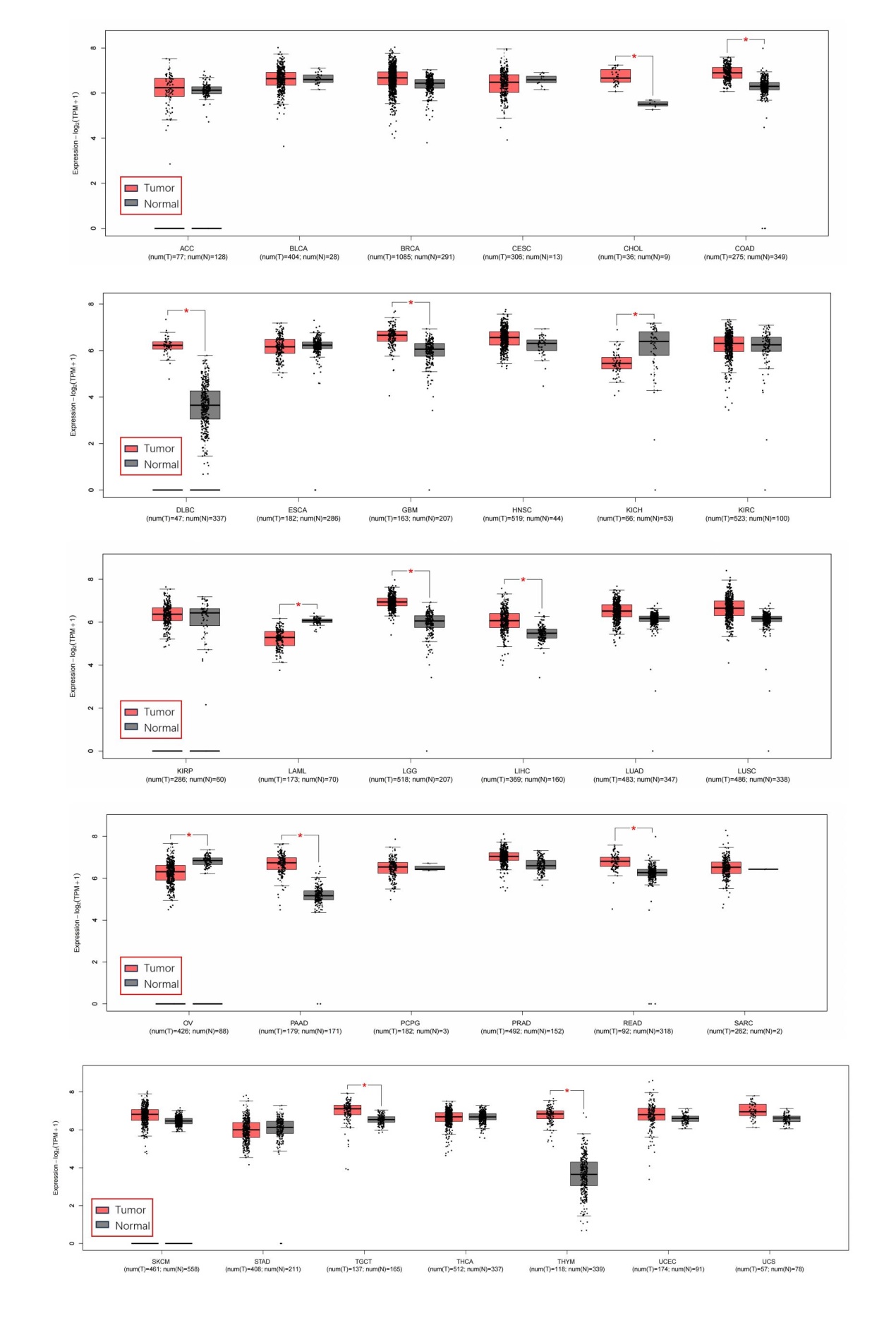
**

**Figure S1. Expression of AAMP in various tumor tissues and corresponding normal tissues.**

Supplement: Supplementary file 1 — Figure S1 [file CCS3-19-e70023-s001.docx]
